# The History of Medical Attendance on the Poor

**Published:** 1922-09

**Authors:** 


### THE HISTORY OF MEDICAL ATTENDANCE ON THE POOR.

AT the annual meeting of the Poor Law Medical Officers' Association, the President, Sir Arthur Newsholme, delivered an interesting address on this subject, which is reported in the *British Medical Journal*. The history of medical attendance on the poor in this country he regarded as tantamount to the history of civilisation. No one test of our distance from barbarism equalled that furnished by the care of the sick poor. He traced the history of the Poor Law service from feudal times to the present day. The Act of Richard II., in 1388, contained the beginnings of the principle of "settlement," which had been so mischievous in preventing the mobility of labour. During the reign of Charles II. in 1662 this Act was renewed and extended. It made labourers cling to their parish under conditions allied to slavery. Prior to the reign of Henry VIII. the wanderings of vagrants had been controlled by terribly severe enactments. The drastic punishments, when no relief agencies existed, were found to provide no remedy. About this time a distinction was drawn between the sick and impotent who were unable to work and the lusty unemployed who could work. In the reign of Elizabeth power was given to the magistrates to levy a tax on voluntary defaulters. Overseers were appointed to provide for the impotent and to provide work in exchange for relief. Thus the coupling of work with relief was established. Towards the end of the seventeenth century the institution of workhouses led to a great reduction in the claims on the poor rate. By Gilbert's Act (1782) the management of poor relief was no longer entirely in the hands of overseers and churchwardens. The administration of the funds was entrusted to the magistrates, who were to appoint visitors and guardians, the latter being paid officials with duties like those of the modern relieving officer. This altered the fundamental principle of the workhouse. In 1796 outdoor relief to the able-bodied was specifically legalised. Following this and similar changes the cost of relief bounded up. In 1834 the public funds were regarded as a regular part of the maintenance of agricultural labourers. These funds were administered in an absolutely uncontrolled manner. Then came the Royal Commission on the Poor Law in 1834, which concluded that the abuses and evils of the existing system were the direct result of indiscriminate outdoor relief to the able-bodied, and the condition was laid down that the pauper's economic situation on the whole shall not be made really or apparently so eligible as the situation of the independent labourer of the lowest classes.

Then followed the formation of larger Poor Law unions, and a rapid reduction of outdoor relief for the able-bodied. The problem became one of old age, and when not old age it was sickness largely. Nursing by paupers was prohibited in 1895. So rapid had been the organisation of medical treatment that at this time there were twice as many beds in Metropolitan Poor Law infirmaries as in the voluntary hospitals, and nearly as many as in all the voluntary hospitals throughout England and Wales. The improvement in the quality of medical attendance and nursing in the best infirmaries had been no less remarkable. It was possible to solve satisfactorily the general hospital problem only by securing more complete co-ordination of the work of the voluntary and special hospitals, thus relieving voluntary hospitals of a large number of chronic and incurable cases. Deterrent administration was legitimate for the able-bodied, though investigation of causes and conditions would often supply a more rational line of action, but for the actually sick the best which medical knowledge and devoted nursing could supply must be given. However the illness had been acquired, it was imperative, not only in the interest of the patient, but also of the community, that treatment should be prompt and adequate, and that no partial definition of what constituted medical treatment should be tolerated.

To realise the full utility of Poor Law work it must be closely linked up with public health work. Poor Law medical officers visiting the homes of the poor, and familiar with their home conditions, became part-time Medical Officers of Health. But the attempt to combine prevention and treatment proved unsuccessful because the Poor Law medical officers visited only a small proportion of the dwellings of the poor, because they were not trained in prevention work, and because the good seed of preventive work was choked by the increasing competition of private medical practice. Ere long it should become practicable for medical practitioners engaged in clinical work, partially or entirely at the public expense, to be on the staff of the local public health authority in the district covered by their practice while carrying on their clinical work, to the great advantage of patients and of the public, as well as of themselves. This would need to be organised in connection with a general service, curative as well as medical. Its realisation, though difficult, presented no insuperable obstacles.

We are requested by the Ministry of Pensions to state that any new claim to pension in respect of disablement under any Warrant, Order in Council, or Order, administered by the Minister of Pensions, must be made within seven years after the claimant was discharged, or before September 1, 1928, *whichever is the earlier.*

